# Natural Transformation in *Acinetobacter baumannii* W068: A Genetic Analysis Reveals the Involvements of the CRP, XcpV, XcpW, TsaP, and TonB_2_

**DOI:** 10.3389/fmicb.2021.738034

**Published:** 2022-01-20

**Authors:** Yuan Hu, Junjie Zheng, Jianzhong Zhang

**Affiliations:** ^1^State Key Laboratory of Infectious Disease Prevention and Control, Collaborative Innovation Center for Diagnosis and Treatment of Infectious Diseases, National Institute for Communicable Disease Control and Prevention, Chinese Center for Disease Control and Prevention, Beijing, China; ^2^The Fifth Medical Center of Chinese PLA General Hospital, Beijing, China

**Keywords:** *Acinetobacter baumannii*, natural transformation, type IV pili, DNA uptake, competence pseudopilus, type II secretion systems

## Abstract

*Acinetobacter baumannii* is a serious threat to public health, and there is increasing attention to the development of antibiotic resistance in this bacterium. Natural transformation is a major horizontal gene transfer mechanism that can lead to antibiotic resistance. To better understand the mechanism of natural transformation in *A. baumannii*, we selected a clinical isolate that was transformable but had no visible extracellular type IV pili (T4P) filaments and then examined the effects of multiple single-gene knockouts on natural plasmid transformation. Among 33 candidate genes, 28 knockout mutants had severely or completely impaired transformability. Some of these genes had established roles in T4P biogenesis; DNA transfer across the outer membrane, periplasm, or inner membrane; and protection of intracellular single-stranded DNA (ssDNA). Other genes had no previously reported roles in natural transformation of *A. baumannii*, including competence activator cAMP receptor protein (CRP), a periplasmic protein that may function in T4P assembly (TonB_2_), a T4P secretin-associated protein (TsaP), and two type II secretion system (T2SS) minor pseudopilus assembly prime complex competent proteins (XcpV and XcpW). The deletion of the T2SS assembly platform protein X had no effect on transformation, and the minor pseudopilins were capable of initiating major pilin assembly. Thus, we speculate that XcpV and XcpW may function in DNA uptake with major pilin assembly, a non-T2SS-dependent mechanism and that a competence pseudopilus similar to T4P constituted the central part of the DNA uptake complex. These results may help guide future research on the alarming increase of antibiotic resistance in this pathogen.

## Introduction

*Acinetobacter baumannii* is a Gram-negative coccobacillus that is a significant public health concern worldwide because of its remarkable ability to develop antibiotic resistance (Peleg et al., 2008). Horizontal gene transfer (HGT) is mostly responsible for the alarming development of resistance in this species (Snitkin et al., 2011). There are three main mechanisms of HGT in bacteria: transduction, conjugation, and natural transformation. Natural transformation does not rely on mobile genetic elements but is driven solely by a developmental program in the recipient bacteria. It begins with the triggering of natural competence in recipient bacteria, a physiological state that allows them to take up genetic material from their surroundings. Then, external DNA enters the bacteria and recombines into the DNA of the chromosome or reconstitutes the plasmid within the recipient (Chen and Dubnau, 2004). Natural transformation is widespread, and, approximately, 80 bacterial species are known to undergo natural transformation (Johnston et al., 2014), although only a few of these species have been intensively studied. Most of our knowledge of natural transformation is from studies of several model organisms, such as *Vibrio cholerae* (Matthey and Blokesch, 2016).

Development of the state of natural competence is tightly controlled by organism-specific processes (MacFadyen et al., 2001; Claverys and Havarstein, 2002; Hamoen et al., 2003). There are major differences among different species, but most species use similar proteins to import DNA, with a notable exception of *Helicobacter pylori* (Chen and Dubnau, 2004). The major components of this competence system are proteins that function in the assembly of type IV pili (T4P) and type II secretion systems (T2SSs) (Seitz and Blokesch, 2013a).

Type II secretion system and T4P are in a widespread superfamily of membrane nanomachines that specialize in the assembly of dynamic helical fibers from the plasma membrane localized pilin subunits in a conserved pattern (Berry and Pelicic, 2015; Leighton et al., 2015; Thomassin et al., 2017). T4P are thin and flexible filaments on the surface of bacteria, but fibers of T2SS are short and remain periplasmic under normal physiological conditions and are referred to as pseudopili (Sandkvist, 2001). The T4P and T2SS assembly system are similar in composition and structure (McLaughlin et al., 2012), and a complex consisting of minor subunits of each system function as primers of each system (Cisneros et al., 2012a; Nguyen et al., 2015; Jacobsen et al., 2020). After being processed by a dedicated prepilin peptidase, the mature pilins/pseudopilins polymerized into helical fibers in conjunction with the inner membrane assembly platform protein, the alignment subcomplex, and motor ATPases (Leighton et al., 2015; Thomassin et al., 2017).

The key process of DNA uptake is as follows: ComEA, the periplasmic DNA-binding protein, pulls external DNA into the periplasm through the outer membrane pilQ secretion. Then, after degradation of one strand, the single-stranded DNA (ssDNA) crosses the inner membrane *via* ComA (ComEC in some species) channel, with the assistance of ComF (Dubnau and Blokesch, 2019). Once this exogenous DNA is inside the cytosol, where Ssb and DprA provide protection, it undergoes incorporation into host’s genome by homologous recombination *via* RecA (Mortier-Barriere et al., 2007), illegitimate recombination or transposition (Hulter and Wackernagel, 2008; Kloos et al., 2021).

Type IV pili are retractable fibers that dynamically polymerize and depolymerize pilins (driven by two ATPases, PilB, and PilT). This enables T4P to pull bacteria along a semi-solid medium (twitching motility) and pull bound substrates such as DNA into the periplasm. This led to the hypothesis that DNA uptake might be a side effect of the T4P-mediated twitching motility (Bakkali, 2013).

However, our preliminary observations indicated that many transformable clinical strains (14.5%, 8/55) of *A. baumannii* had no twitching motility and no detectable T4P based on transmission electron microscopy (data not shown). This led us to question whether T4P is still necessary for them to take up DNA by natural transformation. We therefore selected the strain *A. baumannii* W068 and constructed single-gene knockout mutants by targeting a set of candidate genes that presumably functioned in natural transformation, on the basis of studies of other naturally competent bacteria. Our purpose was to identify genes that functioned in natural competence and are required for the efficient natural transformation of this bacterium and to provide further insight into the DNA uptake process of this new member of the transformable species.

## Materials and Methods

### Bacterial Strains and Growth Conditions

All strains are derivatives of the wild-type clinical isolate *A. baumannii* W068, which is isogenic to the fully sequenced D1279779 (Farrugia et al., 2013). This strain was screened from a survey of natural transformation ability of clinical *A. baumannii* strains (Hu et al., 2019) but had no twitching motility. Bacteria were grown in lysogeny broth (LB). For selection, the growth medium was supplemented with tetracycline (10 μg/ml), kanamycin (50 μg/ml), or zeocin (250 μg/ml). Bacteria were electrotransformed according to the following parameters: 1800 V, 200 Ω, and 25 μF.

### Construction of Plasmids and Mutant Strains

For the generation of deletion strains, a *sacB* gene for sucrose selection amplified from pWM91 was cloned into pGEM-T, resulting in a counter-selectable suicide vector pGEM-sacB. *A. baumannii* mutants were constructed using a standard allelic exchange approach with integrative plasmids on the basis of pGEM-sacB. PCR-amplified fragments of the flanking regions of the desired genetic regions, the kanamycin cassette selection marker, and the suicide vector pGEM-sacB were joined using a seamless cloning strategy on the basis of overlap extension PCR (*pEASY*^®^-Basic Seamless Cloning and Assembly Kit, TRAN). The selection marker kanamycin cassette was flanked by upstream and downstream regions. The correct cloning products were screened by colony PCR of the *E. coli* transformants with primers located in pGEM-sacB (plasmid-F and plasmid R) and confirmed by sequencing. Each constructed plasmid was electroporated into *A. baumannii* W068, and the resulting transformants were selected on kanamycin-containing plates. The correct single-crossover recombinations were screened by colony PCR with primers located in the target gene and the vector sequence. Then, the transformants were counter-selected on LB agar that was supplemented with 10% sucrose agar and kanamycin (50 μg/ml) for the final knockout mutants. Deletion of target genes was verified by colony PCR. Supplementary Table 1 lists the plasmids and *A. baumannii* strains used in this study, and Supplementary Table 2 lists all the primers used in this study.

To ensure that each deletion mutation was not polar on the downstream gene, primers used for deletion of the target gene were designed to preserve at least 30 nucleotides in the 5′ region of the flanking gene, so the ribosome-binding site remained intact.

For complementation of each gene knockout mutant, the PCR product of the full-length gene and about 800 bp of the upstream sequence, and the tetracycline cassette PCR product (from pWH1266) were cloned into pGEM-T plasmid using seamless cloning. Then, these complement plasmids were electroporated into the corresponding knockout mutants, and the complemented mutants were then selected on kanamycin- and tetracycline-containing plates. Correct insertion of the complement plasmid into the genome was verified by PCR and sequencing.

### Natural Transformation Assay

Bacteria were tested for natural transformation as described previously (Hu et al., 2019). The donor DNA pOri was a shuttle-plasmid constructed by cloning the PCR product of the replication origin region of pWH1266 into pCR-Blunt II-TOPO. The zeocin resistance cassette of pCR-Blunt II-TOPO was used as the selectable marker. All experiments were performed at least three times, and statistical analysis was performed by SPSS24. The differences in transformation frequencies were considered significant when *P*-values from Welch’s *t*-test on log-transformed data were below 0.05 (*) or 0.01 (**).

## Results

### Null Mutations in Candidate Genes

**FIGURE 1:**
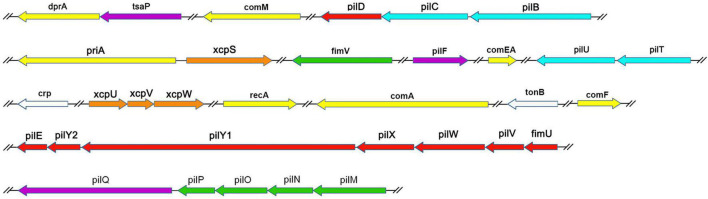
Genetic organization of *A. baumannii* transformation-related homologs examined in this study. Each gene is colored based on the general function of the encoded protein: purple, T4P outer membrane secretin subcomplex; green, T4P alignment subcomplex; blue, T4P motor subcomplex; red, T4P helical pilus filament-related; white, regulators; yellow, DNA uptake and processing; orange, type II secretion. Line breaks (//) denote non-contiguous genomic organization.

We examined the process of DNA uptake in naturally competent *A. baumannii* W068 by selecting a series of candidate genes for knockout. We successfully knocked out 33 candidate genes that were distributed among 16 gene clusters (Table 1 and Figure 1). Twenty of these genes are homologous to genes related to T4P biogenesis (purple, green, blue, and red in Figure 1), seven are related to DNA uptake and processing (yellow in Figure 1), and four are related to T2SS (orange in Figure 1). The two remaining genes are *crp*, a competence regulator gene, and *tonB*_2_, which we considered possibly involved in natural transformation. To our knowledge, this is the first study to investigate the possible functions of eight of these genes (*tsaP*, *fimV*, *priA*, *xcpS*, *xcpU*, *xcpV*, *xcpW*, and *tonB*_2_) in natural transformation.

**TABLE 1:** Genes with potential roles in the natural transformation of *A. baumannii*.

Gene	Locus tags^#^ (ABD1_XXXXX)	Gene products	Homologs^$^			Transformation effect of null mutations^&^			
			*P. aeruginosa* PAO1 (PAXXXX)	*V. cholerae* N16961 (VCXXXX)	*A. baylyi* ADP1 (ACIADXXXX)	*A. baumannii* W068	*V. cholerae* N16961	*A. baylyi* ADP1	*A. baumannii* A118
**T4P outer membrane secretin subcomplex**									
pilF	04710	Type IV pilus biogenesis protein	pilF (3805)	pilF (1612)	pilF (0558)	<d.l.	<d.l.	<d.l.	
pilQ	30760	Fimbrial assembly protein PilQ	pilQ (5040)	pilQ (2630)	comQ (3355)	<d.l.	<d.l.	<d.l.	<d.l.
tsaP	01670	LysM peptidoglycan-binding domain-containing protein	PA0020	VC0047	ACIAD0210	↓			
**T4P alignment subcomplex**									
pilM	30800	Type IV pilus assembly protein PilM	pilM (5044)	pilM (2634)	comM (3360)	<d.l.	<d.l.	<d.l.	
pilN	30790	Type IV pilus assembly protein PilN	pilN (5043)	pilN (2633)	comN (3359)	<d.l.	<d.l.		
pilO	30780	Type IV pilus assembly protein PilO″	pilO (5042)	pilO (2632)	comO (3357)	<d.l.	<d.l.		
pilP	30770	Type IV pilus assembly protein PilP	pilP (5041)	pilP (2631)	comL (3356)	<d.l.	<d.l.	<d.l.	
fimV	03970	Hypothetical protein	fimV (3115)	–	–	∼			
**T4P motor subcomplex**									
pilB	03050	Type IV fimbrial assembly ATPase	pilB (4526)	pilB (2424)	pilB (0362)	<d.l.	<d.l.		
pilC	03040	Type IV fimbrial assembly protein	pilC (4527)	PilC (2425)	pilC (0361)	<d.l.	<d.l.	<d.l.	
pilT	08430	Twitching motility protein	pilT (0395)	pilT (0462)	pilT (0912)	<d.l.	<d.l.	<d.l.	<d.l.
pilU	08420	Twitching motility protein	pilU (0396)	pilU (0463)	pilU (0911)	∼	∼	<d.l.	∼
**T4P helical pilus filament**									
pilD	03030	Type IV prepilin peptidase	pilD (4528)	PilD (2426)	pilD (0360)	<d.l.			
pilE	30500	Pilin like competence factor	pilE (4556)	pilE (0857)	comF (3314)	<d.l.	↓	↓	
pilY2	30510	Pilin like competence factor	pilY2 (4555)	–	comE (3315)	<d.l.		∼	
pilY1	30520	Pilus assembly protein tip-associated adhesin PilY1	pilY1 (4554)	–	comC (3316)	<d.l.		<d.l.	
pilX	30530	Pilus assembly protein PilX	pilX (4553)	–	pilX (3317)	<d.l.		<d.l.	
pilW	30540	Pilus assembly protein PilW	pilW (4552)	–	comB (3318)	<d.l.		<d.l.	
pilV	30550	Type IV pilus modification protein PilV	pilV (4551)	–	pilV (3319)	<d.l.		<d.l.	
fimU	30560	Pilin protein	fimU (4550)	–	fimU (3321)	↓		∼	
**DNA uptake and processing**									
comEA	05880	Putative late competence protein ComEA, DNA receptor	PA3140	comEA (1917)	comEA (3064)	<d.l.	<d.l.	↓	<d.l.
comA	25630	Competence factor involved in DNA uptake	PA2984	comEC (1879)	comA (2639)	<d.l.	<d.l.	<d.l.	
comF	29810	DNA transformation protein ComF	PA0489	comF (2719)	comF (3236)	<d.l.	<d.l.		<d.l.
priA	03400	Helicase essential for oriC/DnaA-independent DNA replication	priA (5050)	priA (2678)	priA (0409)	<d.l.			
dprA	01660	Hypothetical protein	PA0021	VC0048	ACIAD0209	↓			<d.l.
recA	19880	Recombinase A	recA (3617)	recA (0543)	recA (1385)	<d.l.	<d.l.		
comM	02020	Competence protein ComM	PA5290	VC0032	comM (0242)	∼			∼
**Type II secretion system**									
xcpS	03410	General secretion pathway protein F	xcpS (3102)	gspF (2731)	xcpS (0411)	∼			
xcpU	15710	General secretion pathway protein G precursor	xcpU (3010)	gspH (2729)	ACIAD2357	∼			
xcpV	15720	General secretion pathway protein I	xcpV (3099)	gspI (2728)	xcpV (2356)	<d.l.			
xcpW	15730	Type II secretion system minor pseudopilin GspJ	xcpW (3098)	gspJ (2727)	xcpW (2355)	<d.l.			
**Others**									
crp	11920	Cyclic AMP receptor Protein	vfr (0652)	crp (2614)	vfr (1262)	<d.l.			
tonB_2_	29160	TonB-dependent receptor	tonB3 (0406)	–	ACIAD1588	↓			

*^#^Locus tags and gene products are according to accession number CP003967.1.*

*^$^Homologs were determined using tblastn.*

*Locus tags of strain PAO1 (PAXXX) are according to accession number NC002516.*

*Locus tags of strain ADP1 (ACIADXXXX) are according to accession number NC005966.*

*Locus tags of strain N16961 [VC(A)XXXX] are according to accession number NC002505.*

*^&^<d.l., below detection limit; ∼, no significant difference from wild-type strain; **↓**, significantly impaired.*

### Natural Transformability of Mutations

**FIGURE 2:**
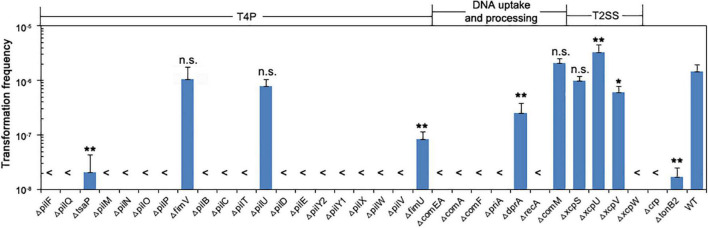
Natural transformation of strains lacking individual competence proteins. Bars show the average transformation frequencies of at least three independent experiments. Error bars indicate SD. Statistical analysis was performed using one-way ANOVA with Dunnett’s multiple comparisons test. **P < 0.01; *P < 0.05; n.s., not significant. Each mutant strain was compared with the wild-type. <, below detection limit.

We examined the 33 knockout mutants by comparing their transformation efficiency with wild-type W068. No knockout strains were affected in growth, but 28 of these mutants were severely or completely impaired in natural transformability (Table 1 and Figure 2). These mutations were in 18 genes related to T4P (*pilF*, *pilQ*, *tsaP*, *pilM*, *pilN*, *pilO*, *pilP*, *pilB*, *pilC*, *pilT*, *pilD*, *pilE*, *pilY2*, *pilY1*, *pilX*, *pilW*, *pilV*, and *fimU*), six genes related to DNA uptake and processing (*comEA*, *comA*, *comF*, *priA*, *dprA*, and *recA*), two genes related to T2SS (*xcpV* and *xcpW*), and the *crp* and *tonB*_2_ genes.

A comparison of our results with previous results from the literature indicated that most of the individual effects that we observed were consistent with those reported in other well-studied transformable bacterial species (Seitz and Blokesch, 2013b; Leong et al., 2017; Vesel and Blokesch, 2021), except for knockouts that had mutations in the *pilU*, *pilE*, *pilY2*, *fimU*, *comEA*, and *dprA* genes (Table 1). In particular, the null mutation of *pilE* led to impaired transformation in *V. cholerae* N16961 and *A. baylyi* ADP1 but led to no transformation in W068. Considering that the transformation efficiencies of the wild-types of these two strains were much higher than that of W068, it seems likely that the effects of knockout were consistent among all strains. Similarly, the null mutation of *comEA* led to impaired transformation in the strain with the highest transformation efficiency (*A. baylyi* ADP1), but transformation was below the detection limits in *V. cholerae* N1696 and in *A. baumannii* A118 and W68.

Type IV pili motor ATPase PilU is dispensable for successful plasmid transformation in W068, consistent with previous results from *V. cholerae* and *A. baumannii* A118, but inconsistent with results from *A. baylyi* (Table 1; Seitz and Blokesch, 2013b; Leong et al., 2017; Vesel and Blokesch, 2021). Deletions of the minor pilin *pilY2* and *fimU* had deleterious effects on the natural competence of W068, also inconsistent with studies of *A. baylyi* (Leong et al., 2017). Therefore, there appear to be slight differences in the mechanisms of natural transformation in *A. baumannii* and the model bacterium *A. baylyi*.

DprA is a ssDNA binding protein previously shown to be essential for the transformation of chromosomal DNA in *A. baumannii* A118 (Vesel and Blokesch, 2021), but in *A. baumannii* W068, Δ*dprA* only led to decreased transformation frequency (mean ± SD: 2.55 × 10^–7^ ± 1.36 × 10^–7^ vs. 1.48 × 10^–6^ ± 4.62 × 10^–7^, *P* = 0.008, Figure 2), indicating that dprA is partially required for plasmid transformation in this strain.

Among the eight genes whose role in transformation we examined for the first time, five were required for the natural transformation of *A. baumannii* W068. Compared with the transformation frequency of the wild-type (mean ± SD: 1.48 × 10^–6^ ± 4.62 × 10^–7^), deletion of XcpW and PriA led to no detectable natural transformation. However, there was impaired transformation in strains with Δ*tsaP* (2.1 × 10^–8^ ± 2.24 × 10^–9^, *P* = 0.004), Δ*xcpV* (6.03 × 10^–7^ ± 1.97 × 10^–7^, *P* = 0.04), and Δ*tonB*_2_ (1.73 × 10^–8^ ± 8.41 × 10^–9^, *P* = 0.004). Deletion of *fimV* and *xcpS* had no impact on competence, and Δ*xcpU* surprisingly increased competence (3.32 × 10^–6^ ± 1.23 × 10^–6^, *P* < 0.001, Figure 2).

### Genetic Complementation of Mutants

We confirmed these results by performing genetic complementation experiments. To obtain efficient and stable transformation, we selected a shuttle plasmid as donor DNA for all transformation assays because the traditional complementation assays based on plasmids may introduce interference due to plasmid incompatibility. We constructed complement mutations using a pGEM-T-based suicide plasmid, in which each gene of interest was reinserted into its original position by recombination. All the complementation assays led to statistically significant restoration of natural transformation, although some did not provide transformation to the full level present in wild-type (Table 2). Natural transformation is a complex process that requires precise coordination of the expression of multiple proteins. We therefore speculate that the foreign sequences introduced in some of these *trans*-complementation assays may have disrupted the coordinated expression of these proteins, leading to slightly impaired transformation.

**TABLE 2:** *Trans*-complementation of *A. baumannii* mutants.

Gene	Transformation frequency		Significance
	Deletion mutant^#^	Complementation	
pilF	<d.l.	8.30 × 10^–7^ ± 4.97 × 10^–8^	**
pilQ	<d.l.	7.57 × 10^–7^ ± 1.20 × 10^–8^	**
tsaP	2.1 × 10^–8^ ± 2.24 × 10^–9^	4.19 × 10^–7^ ± 2.45 × 10^–7^	*
pilM	<d.l.	5.30 × 10^–7^ ± 9.63 × 10^–8^	**
pilN	<d.l.	6.87 × 10^–7^ ± 9.84 × 10^–8^	**
pilO	<d.l.	9.27 × 10^–7^ ± 5.25 × 10^–8^	**
pilP	<d.l.	7.37 × 10^–7^ ± 1.36 × 10^–7^	**
pilB	<d.l.	6.47 × 10^–7^ ± 1.24 × 10^–7^	**
pilC	<d.l.	7.37 × 10^–7^ ± 1.10 × 10^–7^	**
pilT	<d.l.	8.53 × 10^–7^ ± 1.87 × 10^–7^	**
pilD	<d.l.	8.22 × 10^–7^ ± 2.08 × 10^–7^	**
pilE	<d.l.	1.44 × 10^–6^ ± 1.70 × 10^–7^	**
pilY2	<d.l.	7.25 × 10^–7^ ± 4.12 × 10^–8^	**
pilY1	<d.l.	3.92 × 10^–7^ ± 1.16 × 10^–7^	**
pilX	<d.l.	7.83 × 10^–7^ ± 1.02 × 10^–7^	**
pilW	<d.l.	6.43 × 10^–7^ ± 1.14 × 10^–7^	**
pilV	<d.l.	3.85 × 10^–7^ ± 6.97 × 10^–8^	**
fimU	8.45 × 10^–8^ ± 3.11 × 10^–8^	7.63 × 10^–7^ ± 9.67 × 10^–8^	**
crp	<d.l.	5.39 × 10^–7^ ± 9.87 × 10^–8^	**
tonB_2_	1.73 × 10^–8^ ± 8.41 × 10^–9^	6.77 × 10^–7^ ± 1.60 × 10^–7^	*
comEA	<d.l.	6.27 × 10^–7^ ± 1.16 × 10^–7^	**
comA	<d.l.	8.63 × 10^–7^ ± 1.24 × 10^–7^	**
comF	<d.l.	7.27 × 10^–7^ ± 2.53 × 10^–7^	**
priA	<d.l.	8.67 × 10^–7^ ± 1.25 × 10^–7^	**
recA	<d.l.	8.20 × 10^–7^ ± 1.59 × 10^–7^	**
xcpW	<d.l.	7.90 × 10^–7^ ± 1.51 × 10^–7^	**

*^#^<d.l., below detection limit (1.84 × 10^–9^ ± 5.67 × 10^–10^).*

*P < 0.05 (Welch’s t-test); **P < 0.01 (Welch’s t-test).

## Discussion

### Type IV Pili Components Are Required for Natural Transformation in *Acinetobacter baumannii* W068

It is well known that T4P plays an important role in the natural transformation of many bacteria, although the transformable strain that we studied (*A. baumannii* W068) lacks functional T4P. We examined 20 genes related to T4P, but only two of these genes had no impact on competence. These results suggest that, although W068 has no visible extracellular T4P filaments and no twitching motility, the T4P components are still crucial for natural transformation.

Previous studies of *Pseudomonas aeruginosa* indicated that, after being processed by the prepilin peptidase PilD, the mature major pillin subunit (PilA), minor pillin subunits (FimU, PilV, PilW, PilX, and PilE), and two non-pilin proteins (PilY1 and pilY2) were assembled into filaments and functioned in conjunction with an inner membrane assembly platform protein (PilC), the alignment subcomplex (PilM, PilN, PilO, and PilP), and motor ATPases (PilT, PilB, and PilU) (Leighton et al., 2015; Nguyen et al., 2015). In addition, minor pilins PilV-W-X and PilY1 appeared to form an inner membrane subcomplex that acted as a primer for pilus assembly by interacting with PilA *via* PilE and FimU (Nguyen et al., 2015). *P. aeruginosa* cells are not piliated if they are missing even a single protein of the primer subcomplex, but assembly of surface pili can be initiated in the absence of FimU (Nguyen et al., 2015). In contrast, our study of *A. baumannii* W068 indicated that all the minor pilin knockout mutants except Δ*fimU* could not be transformed, and except PilU, all the T4P assembly proteins mentioned above were required for natural transformation. Therefore, we speculate that, although *A. baumannii* W068 has no visible extracellular T4P filaments, the intracellular part of these filaments was still assembled and may form a “pseudopilin” that functioned in natural transformation but cannot be visualized because of their small size.

An outer membrane channel formed by secretin pilQ with the pilotin protein PilF is needed for the pilus to extrude and provide entry to external DNA so that it can enter the periplasmic space (Koo et al., 2013). This PilQ channel is thus “open” when the pili are present and “closed” when the pili are absent (Gold et al., 2015). We therefore speculate that assembly of the intracellular part of the T4P filaments may open the pilQ channel, a necessary condition for DNA uptake.

Previous research suggested that the T4P secretin-associated protein (TsaP) anchored the secretin complex to the peptidoglycan (Siewering et al., 2014). However, deletion of TsaP in *P. aeruginosa* had no effect on the expression or function of T4P (Koo et al., 2016). In contrast, we found that deletion of TsaP led to significantly impaired transformation in *A. baumannii* W068. TsaP in *A. baumanni* also has a peptidoglycan-binding LysM motif, suggesting it may have the same function as in the prototypical system. Therefore, TsaP deletion may affect PilQ formation and thereby disrupt DNA passage through the outer membrane and/or peptidoglycan layer.

Another LysM motif-containing protein in *P. aeruginosa* (FimV) also functions in secretin formation and was supposed to be included in the PilMNOP alignment subcomplex (Leighton et al., 2015). However, we found that Δ*fimV* had no effect on transformation of *A. baumannii* W068; in contrast, each of the PilMNOP alignment subcomplex deletions abrogated *A. baumanni* transformation, consistent with the findings in *V. cholerae* (Seitz and Blokesch, 2013b).

### Type II Secretion System Pseudopilins Are Required for Natural Transformation in *Acinetobacter baumannii* W068

The T2SS has three minor pseudopilins (GspI, GspJ, and GspK) that form a complex equivalent to the T4P PilV-W-X and function as a structural template that promotes initiation of pseudopilus assembly (Cisneros et al., 2012a; Jacobsen et al., 2020). We found that deletion of homologs of GspI (XcpV) or GspJ (XcpW) led to partially or completely impaired natural transformation in *A. baumannii* W068 (Figure 2). However, the role of the T2SS pseudopilus in the natural transformation of *A. baumannii* is still doubtful, because the deletion of the assembly platform protein XcpS (homolog of GspF), the T2SS PilC homolog, had no impact on competence. Previous research indicated that XcpS^GspF^ was required for T2SS pseudopilus formation (Sauvonnet et al., 2000). Thus, the roles of XcpV^GspI^ and XcpW^GspJ^ in DNA uptake must be independent of the T2SS pseudopilus. We suggest that part of the coding products of *xcpV* and *xcpW* may affect a different structure that is also related to the transformation apparatus and functions in the uptake or transport of DNA. In fact, we found that deletion of the fourth minor pseudopilin (XcpU, homolog of GspH) led to increased competence (Figure 2). XcpU^GspH^ is thought to act as a structural linker between the T2SS GspK-I-J complex and the major pseudopilin GspG (Korotkov and Hol, 2008). Therefore, knockout of XcpU^GspH^ may release more XcpV^GspI^ and XcpW^GspJ^ and thus facilitate DNA translocation.

Cisneros et al. (2012b) reported that the minor pilins and minor pseudopilins are functionally interchangeable in initiating major pilin assembly. Therefore, we speculate that XcpV and XcpW may function in DNA uptake by participating in major pilin assembly and formation of a special competence pseudopilus by using the same components as T4P. Chen and Dubnau (2004) proposed the existence of a competence pseudopilus as a structure distinct from T4P, and the minor pilins determine which structure is formed. We agree with these interpretations and propose that the T2SS minor pseudopilins also have a role. We expect the future studies will examine the underlying structures and dynamics of the DNA absorption process of *A. baumannii*.

### DNA Translocation and Processing Proteins Required for Plasmid Transformation in *Acinetobacter baumannii* W068

The DNA-binding competence protein ComEA (which potentially drives DNA into the periplasm), the inner-membrane channel proteins ComA, and a cytoplasmic protein ComF are considered the main components of the DNA uptake machinery (Seitz and Blokesch, 2013b; Dubnau and Blokesch, 2019). This is consistent with our observations that no transformation occurred in strains lacking comEA, comA, or comF. A previous study of *V. cholerae* suggested that an ATP-dependent DNA helicase (PriA) was responsible for pulling DNA through the ComA channel by unwinding dsDNA (Matthey and Blokesch, 2016), but this requires experimental verification. Nonetheless, our Δ*PriA* mutant did not undergo transformation (Figure 2), so this protein is required for the natural transformation of *A. baumannii* W068.

Following the entry into the cytosol, ssDNA must be protected prior to recombination into the recipient genome or reconstitution into a plasmid. DrpA is a ssDNA binding protein previously shown to be essential for transformation of chromosomal DNA in *A. baumannii* A118 (Vesel and Blokesch, 2021); however, for plasmid transformation in W068, our Δ*dprA* mutant only had decreased transformation. Studies of other species indicated that the inactivation of *dprA* decreased chromosomal but not plasmid transformation in *Haemophilus influenzae* (Karudapuram et al., 1995), decreases both chromosomal and plasmid transformation in *Bacillus subtilis* (Tadesse and Graumann, 2007), and eliminates both of them in *Streptococcus pneumoniae* (Berge et al., 2003). Although there were some methodological differences among these studies, the function of DprA in natural transformation appears to be strain-dependent.

In general, non-homologous plasmid transformation is a RecA-independent event, but we found that deletion of recA abolished the plasmid transformation in *A. baumannii* W068. Previous research reported that RecA was required for plasmid transformation in *S. pneumoniae* (Martin et al., 1995) and that the absence of RecA led to rapid degradation of ssDNA (Berge et al., 2003). If RecA_Aba_ has a similar protective role on ssDNA, then it is possible that the incoming ssDNA receives more protection from RecA than DprA. In addition, a recent report found that RecA was required for surface-associated motility, chemotaxis, and the full virulence of *A. baumannii* (Corral et al., 2020). These results suggest that RecA is a multifunctional protein in *A. baumannii*, in that it functions in homologous recombination and in the bacterial surface appendages. This may explain the requirement for RecA in the plasmid transformation.

### Other Proteins Contribute to Natural Transformation in *Acinetobacter baumannii* W068

ComM is a hexameric helicase that promotes branch migration during natural transformation in diverse Gram-negative species (Nero et al., 2018). Many multiple drug-resistant strains of *A. baumannii* have a genomic island named AbaR, which has great diversity in gene content and contains multiple putative antibiotic resistance genes, that is inserted in the comM gene (Hamidian and Hall, 2018), and recent research reported that curing of AbaRs restored the high level of natural transformability (Godeux et al., 2020). However, a Δ*comM* mutant had no defects in transformation in our studies and in a recent study of chromosomal transformation in *A. baumannii* A118 (Vesel and Blokesch, 2021). ComM is therefore not necessary for the natural transformation of *A. baumannii*.

Compared to the DNA uptake complexes, less is known about the initiation of competence. Differences in the conditions that induce competence in different bacterial species result in major differences in the regulatory networks that function in the induction of competence. A global regulator protein, cAMP receptor protein (CRP), is a major shared activator that controls the development of competence in the Pasteurellaceae, Enterobacteriaceae, and Vibrionaceae (Cameron and Redfield, 2006; Lo Scrudato et al., 2014). We found that natural transformation was abolished in the Δ*crp* mutant, indicating its involvement in regulation of competence in *A. baumannii*, although the details of its function require further study.

The TonB-ExbB-ExbD–like energy transduction system is widespread among Gram-negative bacteria. This system transduces the proton motive force to facilitate the active transport of substrates, such as ferric siderophores, hemin, and heme, through the outer membrane (Eick-Helmerich and Braun, 1989). The *A. baumannii* genome contains three *tonB* genes (*tonB*_1_, *tonB*_2_, and *tonB*_3_), and these have some overlapping and some distinct roles (Zimbler et al., 2013; Runci et al., 2019). In the competence state, our comparative proteome analysis indicated that *tonB*_2_ was the only *tonB* with increased expression (data not shown). Deletion of *tonB*_2_ substantially reduced but did not eliminate transformation in W068. Zimbler et al. (2013) found that *tonB*_2_ was dispensable for ferric iron uptake but plays a role in the ability of *A. baumannii* to bind to fibronectin and to adhere to alveolar epithelial cells by unknown mechanisms. A homolog of TonB_2_ functions in motility and T4P assembly in *P. aeruginosa* (Huang et al., 2004). T4P mainly functions in motility, adhesion, and natural transformation, so we speculate that *A. baumannii* TonB_2_ may also function in T4P assembly based on our finding that the knockout mutant had impaired transformation.

**FIGURE 3:**
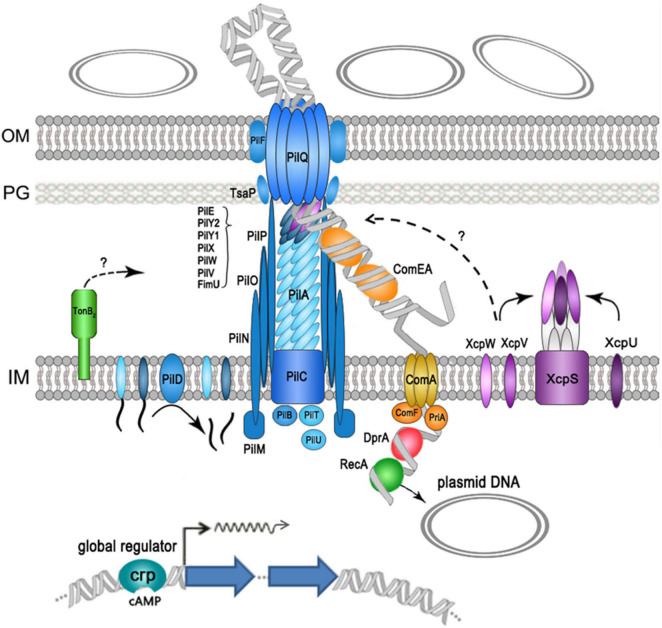
Schematic representation of proteins that function in the natural plasmid transformation in *A. baumannii* W068. Type IV pilus (T4P) components are shown in blue, DNA uptake and translocation proteins are shown in orange, and type II secretion systems (T2SS) proteins are shown in purple.

In summary, our data show that at least 28 genes were required for efficient plasmid transformation in *A. baumannii* W068 (but most likely still incomplete), as summarized in Figure 3. Eighteen of these genes encoded components of T4P, and the others encoded proteins that functioned in DNA translocation (periplasmic DNA-pulling protein ComEA, inner-membrane translocator proteins ComA and ComF, and cytoplasmic DNA-pulling protein PriA) and in protection of the translocated ssDNA (RecA and DprA). We also identified some new proteins that had roles in natural transformation: the competence activator (CRP), a periplasmic protein that may function in T4P assembly (TonB_2_), a TsaP, and two T2SS minor pseudopilins (XcpV and XcpW). Because T4P fiber assembly can also be initiated by minor pseudopilins complex, and the initiation complex may determine which structure forms, we speculate that a competence pseudopilus, which is similar to T4P but does not extend beyond the outer membrane, may account for the main part of the DNA uptake complex in *A. baumannii* W068. Regardless, our results provide unique insight into the natural transformation of *A. baumannii* W068. Whether this competence pseudopilus is responsible for the natural transformation of all strains in this species is uncertain. We suggest that increased research on this topic may be critical for understanding the alarming increase of antibiotic resistance in this emerging pathogen.

## Data Availability Statement

The original contributions presented in the study are included in the article/Supplementary Material, further inquiries can be directed to the corresponding author.

## Author Contributions

JZZ and YH conceived and designed the experiments and drafted the manuscript. YH and JJZ performed the experiments. JJZ performed the statistical analysis. All authors read and approved the final manuscript.

## Conflict of Interest

The authors declare that the research was conducted in the absence of any commercial or financial relationships that could be construed as a potential conflict of interest.

## Publisher’s Note

All claims expressed in this article are solely those of the authors and do not necessarily represent those of their affiliated organizations, or those of the publisher, the editors and the reviewers. Any product that may be evaluated in this article, or claim that may be made by its manufacturer, is not guaranteed or endorsed by the publisher.

## References

[B1] Bakkali M. (2013). Could DNA uptake be a side effect of bacterial adhesion and twitching motility? *Arch. Microbiol.* 195 279–289. 10.1007/s00203-013-0870-1 23381940 PMC3597990

[B2] Berge M. Mortier-Barriere I. Martin B. Claverys J. P. (2003). Transformation of *Streptococcus pneumoniae* relies on DprA- and RecA-dependent protection of incoming DNA single strands. *Mol. Microbiol.* 50 527–536. 10.1046/j.1365-2958.2003.03702.x 14617176

[B3] Berry J. L. Pelicic V. (2015). Exceptionally widespread nanomachines composed of type IV pilins: the prokaryotic Swiss Army knives. *FEMS Microbiol. Rev.* 39 134–154. 10.1093/femsre/fuu001 25793961 PMC4471445

[B4] Cameron A. D. Redfield R. J. (2006). Non-canonical CRP sites control competence regulons in *Escherichia coli* and many other gamma-*proteobacteria*. *Nucleic Acids Res.* 34 6001–6014. 10.1093/nar/gkl734 17068078 PMC1635313

[B5] Chen I. Dubnau D. (2004). DNA uptake during bacterial transformation. *Nat. Rev. Microbiol.* 2 241–249. 10.1038/nrmicro844 15083159

[B6] Cisneros D. A. Bond P. J. Pugsley A. P. Campos M. Francetic O. (2012a). Minor pseudopilin self-assembly primes type II secretion pseudopilus elongation. *EMBO J.* 31 1041–1053. 10.1038/emboj.2011.454 22157749 PMC3280553

[B7] Cisneros D. A. Pehau-Arnaudet G. Francetic O. (2012b). Heterologous assembly of type IV pili by a type II secretion system reveals the role of minor pilins in assembly initiation. *Mol. Microbiol.* 86 805–818. 10.1111/mmi.12033 23006128

[B8] Claverys J. P. Havarstein L. S. (2002). Extracellular-peptide control of competence for genetic transformation in Streptococcus pneumoniae. *Front. Biosci.* 7:d1798–d1814. 10.2741/claverys 12133809

[B9] Corral J. Perez-Varela M. Barbe J. Aranda J. (2020). Direct interaction between RecA and a CheW-like protein is required for surface-associated motility, chemotaxis and the full virulence of *Acinetobacter baumannii* strain ATCC 17978. *Virulence* 11 315–326. 10.1080/21505594.2020.1748923 32255384 PMC7161683

[B10] Dubnau D. Blokesch M. (2019). Mechanisms of DNA uptake by naturally competent bacteria. *Annu. Rev. Genet.* 53 217–237. 10.1146/annurev-genet-112618-043641 31433955

[B11] Eick-Helmerich K. Braun V. (1989). Import of biopolymers into *Escherichia coli*: nucleotide sequences of the exbB and exbD genes are homologous to those of the tolQ and tolR genes, respectively. *J. Bacteriol.* 171 5117–5126. 10.1128/jb.171.9.5117-5126.1989 2670903 PMC210325

[B12] Farrugia D. N. Elbourne L. D. Hassan K. A. Eijkelkamp B. A. Tetu S. G. Brown M. H. (2013). The complete genome and phenome of a community-acquired *Acinetobacter baumannii*. *PLoS One* 8:e58628. 10.1371/journal.pone.0058628 23527001 PMC3602452

[B13] Godeux A. S. Svedholm E. Lupo A. Haenni M. Venner S. Laaberki M. H. (2020). Scarless removal of large resistance Island AbaR results in antibiotic susceptibility and increased natural transformability in *Acinetobacter baumannii*. *Antimicrob. Agents Chemother.* 64 e951–e920. e00951-20, 10.1128/AAC.00951-20 32778544 PMC7508600

[B14] Gold V. A. Salzer R. Averhoff B. Kuhlbrandt W. (2015). Structure of a type IV pilus machinery in the open and closed state. *Elife* 4:e07380. 10.7554/eLife.07380 25997099 PMC4463427

[B15] Hamidian M. Hall R. M. (2018). The AbaR antibiotic resistance islands found in *Acinetobacter baumannii* global clone 1 - structure, origin and evolution. *Drug Resist. Updat.* 41 26–39. 10.1016/j.drup.2018.10.003 30472242

[B16] Hamoen L. W. Venema G. Kuipers O. P. (2003). Controlling competence in *Bacillus subtilis*: shared use of regulators. *Microbiology (Reading)* 149(Pt 1) 9–17. 10.1099/mic.0.26003-0 12576575

[B17] Hu Y. He L. Tao X. Meng F. Zhang J. (2019). High DNA uptake capacity of international clone II *Acinetobacter baumannii* detected by a novel planktonic natural transformation assay. *Front. Microbiol.* 10:2165. 10.3389/fmicb.2019.02165 31616393 PMC6768954

[B18] Huang B. Ru K. Yuan Z. Whitchurch C. B. Mattick J. S. (2004). tonB3 is required for normal twitching motility and extracellular assembly of type IV pili. *J. Bacteriol.* 186 4387–4389. 10.1128/JB.186.13.4387-4389.2004 15205442 PMC421604

[B19] Hulter N. Wackernagel W. (2008). Double illegitimate recombination events integrate DNA segments through two different mechanisms during natural transformation of *Acinetobacter baylyi*. *Mol. Microbiol.* 67 984–995. 10.1111/j.1365-2958.2007.06096.x 18194157

[B20] Jacobsen T. Bardiaux B. Francetic O. Izadi-Pruneyre N. Nilges M. (2020). Structure and function of minor pilins of type IV pili. *Med. Microbiol. Immunol.* 209 301–308. 10.1007/s00430-019-00642-5 31784891 PMC7248040

[B21] Johnston C. Martin B. Fichant G. Polard P. Claverys J. P. (2014). Bacterial transformation: distribution, shared mechanisms and divergent control. *Nat. Rev. Microbiol.* 12 181–196. 10.1038/nrmicro3199 24509783

[B22] Karudapuram S. Zhao X. Barcak G. J. (1995). DNA sequence and characterization of *Haemophilus influenzae* dprA+, a gene required for chromosomal but not plasmid DNA transformation. *J. Bacteriol.* 177 3235–3240. 10.1128/jb.177.11.3235-3240.1995 7768823 PMC177016

[B23] Kloos J. Johnsen P. J. Harms K. (2021). Tn1 transposition in the course of natural transformation enables horizontal antibiotic resistance spread in *Acinetobacter baylyi*. *Microbiology (Reading)* 167:001003. 10.1099/mic.0.001003 33270000 PMC8116780

[B24] Koo J. Lamers R. P. Rubinstein J. L. Burrows L. L. Howell P. L. (2016). Structure of the *Pseudomonas aeruginosa* Type IVa Pilus Secretin at 7.4 A. *Structure* 24 1778–1787. 10.1016/j.str.2016.08.007 27705815

[B25] Koo J. Tang T. Harvey H. Tammam S. Sampaleanu L. Burrows L. L. (2013). Functional mapping of PilF and PilQ in the *Pseudomonas aeruginosa* type IV pilus system. *Biochemistry* 52 2914–2923. 10.1021/bi3015345 23547883

[B26] Korotkov K. V. Hol W. G. (2008). Structure of the GspK-GspI-GspJ complex from the enterotoxigenic *Escherichia coli* type 2 secretion system. *Nat. Struct. Mol. Biol.* 15 462–468. 10.1038/nsmb.1426 18438417

[B27] Leighton T. L. Buensuceso R. N. Howell P. L. Burrows L. L. (2015). Biogenesis of *Pseudomonas aeruginosa* type IV pili and regulation of their function. *Environ. Microbiol.* 17 4148–4163. 10.1111/1462-2920.12849 25808785

[B28] Leong C. G. Bloomfield R. A. Boyd C. A. Dornbusch A. J. Lieber L. Liu F. (2017). The role of core and accessory type IV pilus genes in natural transformation and twitching motility in the bacterium *Acinetobacter baylyi*. *PLoS One* 12:e0182139. 10.1371/journal.pone.0182139 28771515 PMC5542475

[B29] Lo Scrudato M. Borgeaud S. Blokesch M. (2014). Regulatory elements involved in the expression of competence genes in naturally transformable *Vibrio cholerae*. *BMC Microbiol.* 14:327. 10.1186/s12866-014-0327-y 25539806 PMC4299799

[B30] MacFadyen L. P. Chen D. Vo H. C. Liao D. Sinotte R. Redfield R. J. (2001). Competence development by *Haemophilus influenzae* is regulated by the availability of nucleic acid precursors. *Mol. Microbiol.* 40 700–707. 10.1046/j.1365-2958.2001.02419.x 11359575

[B31] Martin B. Garcia P. Castanie M. P. Claverys J. P. (1995). The recA gene of Streptococcus pneumoniae is part of a competence-induced operon and controls lysogenic induction. *Mol. Microbiol.* 15 367–379. 10.1111/j.1365-2958.1995.tb02250.x 7538190

[B32] Matthey N. Blokesch M. (2016). The DNA-uptake process of naturally competent *Vibrio cholerae*. *Trends Microbiol.* 24 98–110. 10.1016/j.tim.2015.10.008 26614677

[B33] McLaughlin L. S. Haft R. J. Forest K. T. (2012). Structural insights into the Type II secretion nanomachine. *Curr. Opin. Struct. Biol.* 22 208–216. 10.1016/j.sbi.2012.02.005 22425326 PMC3341957

[B34] Mortier-Barriere I. Velten M. Dupaigne P. Mirouze N. Pietrement O. McGovern S. (2007). A key presynaptic role in transformation for a widespread bacterial protein: DprA conveys incoming ssDNA to RecA. *Cell* 130 824–836. 10.1016/j.cell.2007.07.038 17803906

[B35] Nero T. M. Dalia T. N. Wang J. C. Kysela D. T. Bochman M. L. Dalia A. B. (2018). ComM is a hexameric helicase that promotes branch migration during natural transformation in diverse Gram-negative species. *Nucleic Acids Res.* 46 6099–6111. 10.1093/nar/gky343 29722872 PMC6158740

[B36] Nguyen Y. Sugiman-Marangos S. Harvey H. Bell S. D. Charlton C. L. Junop M. S. (2015). *Pseudomonas aeruginosa* minor pilins prime type IVa pilus assembly and promote surface display of the PilY1 adhesin. *J. Biol. Chem.* 290 601–611. 10.1074/jbc.M114.616904 25389296 PMC4281761

[B37] Peleg A. Y. Seifert H. Paterson D. L. (2008). *Acinetobacter baumannii*: emergence of a successful pathogen. *Clin. Microbiol. Rev.* 21 538–582. 10.1128/CMR.00058-07 18625687 PMC2493088

[B38] Runci F. Gentile V. Frangipani E. Rampioni G. Leoni L. Lucidi M. (2019). Contribution of active iron uptake to *Acinetobacter baumannii* Pathogenicity. *Infect. Immun.* 87:e00755-18. 10.1128/IAI.00755-18 30718286 PMC6434119

[B39] Sandkvist M. (2001). Biology of type II secretion. *Mol. Microbiol.* 40 271–283. 10.1046/j.1365-2958.2001.02403.x 11309111

[B40] Sauvonnet N. Vignon G. Pugsley A. P. Gounon P. (2000). Pilus formation and protein secretion by the same machinery in *Escherichia coli*. *EMBO J.* 19 2221–2228. 10.1093/emboj/19.10.2221 10811613 PMC384360

[B41] Seitz P. Blokesch M. (2013a). Cues and regulatory pathways involved in natural competence and transformation in pathogenic and environmental gram-negative bacteria. *FEMS Microbiol. Rev.* 37 336–363. 10.1111/j.1574-6976.2012.00353.x 22928673

[B42] Seitz P. Blokesch M. (2013b). DNA-uptake machinery of naturally competent *Vibrio cholerae*. *Proc. Natl. Acad. Sci. U.S.A.* 110 17987–17992. 10.1073/pnas.1315647110 24127573 PMC3816411

[B43] Siewering K. Jain S. Friedrich C. Webber-Birungi M. T. Semchonok D. A. Binzen I. (2014). Peptidoglycan-binding protein TsaP functions in surface assembly of type IV pili. *Proc. Natl. Acad. Sci. U.S.A.* 111 E953–E961. 10.1073/pnas.1322889111 24556993 PMC3956165

[B44] Snitkin E. S. Zelazny A. M. Montero C. I. Stock F. Mijares L. Program N. C. S. (2011). Genome-wide recombination drives diversification of epidemic strains of *Acinetobacter baumannii*. *Proc. Natl. Acad. Sci. U.S.A.* 108 13758–13763. 10.1073/pnas.1104404108 21825119 PMC3158218

[B45] Tadesse S. Graumann P. L. (2007). DprA/Smf protein localizes at the DNA uptake machinery in competent *Bacillus subtilis* cells. *BMC Microbiol.* 7:105. 10.1186/1471-2180-7-105 18045469 PMC2216020

[B46] Thomassin J. L. Santos Moreno J. Guilvout I. Tran Van Nhieu G. Francetic O. (2017). The trans-envelope architecture and function of the type 2 secretion system: new insights raising new questions. *Mol. Microbiol.* 105 211–226. 10.1111/mmi.13704 28486768

[B47] Vesel N. Blokesch M. (2021). Pilus production in *Acinetobacter baumannii* is growth phase dependent and essential for natural transformation. *J. Bacteriol.* 203:e00034-21. 10.1128/JB.00034-21 33495250 PMC8088505

[B48] Zimbler D. L. Arivett B. A. Beckett A. C. Menke S. M. Actis L. A. (2013). Functional features of TonB energy transduction systems of *Acinetobacter baumannii*. *Infect. Immun.* 81 3382–3394. 10.1128/IAI.00540-13 23817614 PMC3754232

