# Supplementary material for: Natural Transformation in Acinetobacter baumannii W068: A Genetic Analysis Reveals the Involvements of the CRP, XcpV, XcpW, TsaP, and TonB2
Source: Front Microbiol. 2022 Jan 20;12:738034. doi: 10.3389/fmicb.2021.738034 (PMC8811193; doi:10.3389/fmicb.2021.738034)
Supplement: Supplementary file 1 [file Table_1.DOCX]

**Table 1S** Plasmids and strains used in this study

| **Plasmids or strains** | **Genotype/description** | **source** |
| --- | --- | --- |
| **Plasmids** |  |  |
| pOri | pCR-Blunt II-TOPO with the replication origin region of pWH1266; Kan^R^, Zeo^R^ | (1) |
| pGEM-T | T vector,Amp^R^ | Promega |
| pGEM-sacB | *sacB* cloned into pGEM-T, suicide plasmid, Amp^R^ | This study |
| pGEM-sacB-△pilF | pilF upstream -kan-pilF downstream cloned into pGEM-sacB; Kan^R^, Amp^R^ | This study |
| pGEM-sacB-△pilQ | pilQ upstream-kan-pilQ downstream cloned into pGEM-sacB; Kan^R^, Amp^R^ | This study |
| pGEM-sacB-△tsaP | tsaP upstream-kan-tsaP downstream cloned into pGEM-sacB; Kan^R^, Amp^R^ | This study |
| pGEM-sacB-△pilM | pilM upstream-kan-pilM downstream cloned into pGEM-sacB; Kan^R^, Amp^R^ | This study |
| pGEM-sacB-△pilN | pilN upstream-kan-pilN downstream cloned into pGEM-sacB; Kan^R^, Amp^R^ | This study |
| pGEM-sacB-△pilO | pilO upstream-kan-pilO downstream cloned into pGEM-sacB; Kan^R^, Amp^R^ | This study |
| pGEM-sacB-△pilP | pilP upstream-kan-pilP downstream cloned into pGEM-sacB; Kan^R^, Amp^R^ | This study |
| pGEM-sacB-△fimV | fimV upstream-kan-fimV downstream cloned into pGEM-sacB; Kan^R^, Amp^R^ | This study |
| pGEM-sacB-△pilB | pilB upstream-kan-pilB downstream cloned into pGEM-sacB; Kan^R^, Amp^R^ | This study |
| pGEM-sacB-△pilC | pilC upstream-kan-pilC downstream cloned into pGEM-sacB; Kan^R^, Amp^R^ | This study |
| pGEM-sacB-△pilT | pilT upstream-kan-pilT downstream cloned into pGEM-sacB; Kan^R^, Amp^R^ | This study |
| pGEM-sacB-△pilU | pilU upstream-kan-pilU downstream cloned into pGEM-sacB; Kan^R^, Amp^R^ | This study |
| pGEM-sacB-△pilD | pilD upstream-kan-pilD downstream cloned into pGEM-sacB; Kan^R^, Amp^R^ | This study |
| pGEM-sacB-△pilE | pilE upstream-kan-pilE downstream cloned into pGEM-sacB; Kan^R^, Amp^R^ | This study |
| pGEM-sacB-△pilY2 | pilY2 upstream-kan-pilY2 downstream cloned into pGEM-sacB; Kan^R^, Amp^R^ | This study |
| pGEM-sacB-△pilY1 | pilY1 upstream-kan-pilY1 downstream cloned into pGEM-sacB; Kan^R^, Amp^R^ | This study |
| pGEM-sacB-△pilX | pilX upstream-kan-pilX downstream cloned into pGEM-sacB; Kan^R^, Amp^R^ | This study |
| pGEM-sacB-△pilW | pilW upstream-kan-pilW downstream cloned into pGEM-sacB; Kan^R^, Amp^R^ | This study |
| pGEM-sacB-△pilV | pilV upstream-kan-pilV downstream cloned into pGEM-sacB; Kan^R^, Amp^R^ | This study |
| pGEM-sacB-△fimU | fimU upstream-kan-fimU downstream cloned into pGEM-sacB; Kan^R^, Amp^R^ | This study |
| pGEM-sacB-△crp | crp upstream-kan-crp downstream cloned into pGEM-sacB; Kan^R^, Amp^R^ | This study |
| pGEM-sacB-△comEA | comEA upstream-kan-comEA downstream cloned into pGEM-sacB; Kan^R^, Amp^R^ | This study |
| pGEM-sacB-△comA | comA upstream-kan-comA downstream cloned into pGEM-sacB; Kan^R^, Amp^R^ | This study |
| pGEM-sacB-△comF | comF upstream-kan-comF downstream cloned into pGEM-sacB; Kan^R^, Amp^R^ | This study |
| pGEM-sacB-△priA | priA upstream-kan-priA downstream cloned into pGEM-sacB; Kan^R^, Amp^R^ | This study |
| pGEM-sacB-△dprA | dprA upstream-kan-dprA downstream cloned into pGEM-sacB; Kan^R^, Amp^R^ | This study |
| pGEM-sacB-△recA | recA upstream-kan-recA downstream cloned into pGEM-sacB; Kan^R^, Amp^R^ | This study |
| pGEM-sacB-△comM | comM upstream-kan-comM downstream cloned into pGEM-sacB; Kan^R^, Amp^R^ | This study |
| pGEM-sacB-△tonB_2_ | tonB_2_ upstream-kan-tonB_2_ downstream cloned into pGEM-sacB; Kan^R^, Amp^R^ | This study |
| pGEM-sacB-△xcpS | xcpS upstream-kan-xcpS downstream cloned into pGEM-sacB; Kan^R^, Amp^R^ | This study |
| pGEM-sacB-△ xcpU | xcpU upstream-kan-xcpU downstream cloned into pGEM-sacB; Kan^R^, Amp^R^ | This study |
| pGEM-sacB-△xcpV | xcpV upstream-kan-xcpV downstream cloned into pGEM-sacB; Kan^R^, Amp^R^ | This study |
| pGEM-sacB-△ xcpW | xcpW upstream-kan-xcpW downstream cloned into pGEM-sacB; Kan^R^, Amp^R^ | This study |
| pGEM-pilF | upstream and pilF – tet cloned into pGEM; Tet^R^, Amp^R^ | This study |
| pGEM-pilQ | upstream and pilQ – tet cloned into pGEM; Tet^R^, Amp^R^ | This study |
| pGEM-tsaP | upstream and tsaP – tet cloned into pGEM; Tet^R^, Amp^R^ | This study |
| pGEM-pilM | upstream and pilM – tet cloned into pGEM; Tet^R^, Amp^R^ | This study |
| pGEM-pilN | upstream and pilN – tet cloned into pGEM; Tet^R^, Amp^R^ | This study |
| pGEM-pilO | upstream and pilO – tet cloned into pGEM; Tet^R^, Amp^R^ | This study |
| pGEM-pilP | upstream and pilP – tet cloned into pGEM; Tet^R^, Amp^R^ | This study |
| pGEM-pilB | upstream and pilB – tet cloned into pGEM; Tet^R^, Amp^R^ | This study |
| pGEM-pilC | upstream and pilC – tet cloned into pGEM; Tet^R^, Amp^R^ | This study |
| pGEM-pilT | upstream and pilT– tet cloned into pGEM; Tet^R^, Amp^R^ | This study |
| pGEM-pilD | upstream and pilD – tet cloned into pGEM; Tet^R^, Amp^R^ | This study |
| pGEM-pilE | upstream and pilE – tet cloned into pGEM; Tet^R^, Amp^R^ | This study |
| pGEM-pilY2 | upstream and pilY2 – tet cloned into pGEM; Tet^R^, Amp^R^ | This study |
| pGEM-pilY1 | upstream and pilY1 – tet cloned into pGEM; Tet^R^, Amp^R^ | This study |
| pGEM-pilX | upstream and pilX – tet cloned into pGEM; Tet^R^, Amp^R^ | This study |
| pGEM-pilW | upstream and pilW – tet cloned into pGEM; Tet^R^, Amp^R^ | This study |
| pGEM-pilV | upstream and pilV – tet cloned into pGEM; Tet^R^, Amp^R^ | This study |
| pGEM-fimU | upstream and fimU – tet cloned into pGEM; Tet^R^, Amp^R^ | This study |
| pGEM-tonB_2_ | upstream and tonB_2_ – tet cloned into pGEM; Tet^R^, Amp^R^ | This study |
| pGEM-crp | upstream and crp – tet cloned into pGEM; Tet^R^, Amp^R^ | This study |
| pGEM-comEA | upstream and comEA – tet cloned into pGEM; Tet^R^, Amp^R^ | This study |
| pGEM-comA | upstream and comA – tet cloned into pGEM; Tet^R^, Amp^R^ | This study |
| pGEM-comF | upstream and comF – tet cloned into pGEM; Tet^R^, Amp^R^ | This study |
| pGEM-priA | upstream and priA– tet cloned into pGEM; Tet^R^, Amp^R^ | This study |
| pGEM-recA | upstream and recA – tet cloned into pGEM; Tet^R^, Amp^R^ | This study |
| pGEM-xcpW | upstream and xcpW – tet cloned into pGEM; Tet^R^, Amp^R^ | This study |
| ***A. baumannii* strains** |  |  |
| W068 | Wild type, sensitive, no twitching motility | (1) |
| △pilF | W068 with ABD1_04710 deleted, using suicide plasmid pGEM-sacB-△pilF; Kan^R^ | This study |
| △pilQ | W068 with ABD1_30760 deleted, using suicide plasmid pGEM-sacB-△pilQ; Kan^R^ | This study |
| △tsaP | W068 with ABD1_01670 deleted, using suicide plasmid pGEM-sacB-△tsaP; Kan^R^ | This study |
| △pilM | W068 with ABD1_30800 deleted, using suicide plasmid pGEM-sacB-△pilM; Kan^R^ | This study |
| △pilN | W068 with ABD1_30790 deleted, using suicide plasmid pGEM-sacB-△pilN; Kan^R^ | This study |
| △pilO | W068 with ABD1_30780 deleted, using suicide plasmid pGEM-sacB-△pilO; Kan^R^ | This study |
| △pilP | W068 with ABD1_30770 deleted, using suicide plasmid pGEM-sacB-△pilP; Kan^R^ | This study |
| △fimV | W068 with ABD1_03970 deleted, using suicide plasmid pGEM-sacB-△fimV; Kan^R^ | This study |
| △pilB | W068 with ABD1_03050 deleted, using suicide plasmid pGEM-sacB-△pilB; Kan^R^ | This study |
| △pilC | W068 with ABD1_03040 deleted, using suicide plasmid pGEM-sacB-△pilC; Kan^R^ | This study |
| △pilT | W068 with ABD1_08430 deleted, using suicide plasmid pGEM-sacB-△pilT; Kan^R^ | This study |
| △pilU | W068 with ABD1_08420 deleted, using suicide plasmid pGEM-sacB-△pilU; Kan^R^ | This study |
| △pilD | W068 with ABD1_03030 deleted, using suicide plasmid pGEM-sacB-△pilD; Kan^R^ | This study |
| △pilE | W068 with ABD1_30500 deleted, using suicide plasmid pGEM-sacB-△pilE; Kan^R^ | This study |
| △pilY2 | W068 with ABD1_30510 deleted, using suicide plasmid pGEM-sacB-△pilY2; Kan^R^ | This study |
| △pilY1 | W068 with ABD1_30520 deleted, using suicide plasmid pGEM-sacB-△pilY1; Kan^R^ | This study |
| △pilX | W068 with ABD1_30530 deleted, using suicide plasmid pGEM-sacB-△pilX; Kan^R^ | This study |
| △pilW | W068 with ABD1_30540 deleted, using suicide plasmid pGEM-sacB-△pilW; Kan^R^ | This study |
| △pilV | W068 with ABD1_30550 deleted, using suicide plasmid pGEM-sacB-△pilV; Kan^R^ | This study |
| △fimU | W068 with ABD1_30560 deleted, using suicide plasmid pGEM-sacB-△fimU ; Kan^R^ | This study |
| △crp | W068 with ABD1_11920 deleted, using suicide plasmid pGEM-sacB-△crp ; Kan^R^ | This study |
| △comEA | W068 with ABD1_05880 deleted, using suicide plasmid pGEM-sacB-△comEA ; Kan^R^ | This study |
| △comA | W068 with ABD1_25630 deleted, using suicide plasmid pGEM-sacB-△comA ; Kan^R^ | This study |
| △comF | W068 with ABD1_29810 deleted, using suicide plasmid pGEM-sacB-△comF; Kan^R^ | This study |
| △priA | W068 with ABD1_03400 deleted, using suicide plasmid pGEM-sacB-△priA; Kan^R^ | This study |
| △dprA | W068 with ABD1_01660 deleted, using suicide plasmid pGEM-sacB-△dprA; Kan^R^ | This study |
| △recA | W068 with ABD1_19880 deleted, using suicide plasmid pGEM-sacB-△recA; Kan^R^ | This study |
| △comM | W068 with ABD1_02020 deleted, using suicide plasmid pGEM-sacB-△comM ; Kan^R^ | This study |
| △tonB_2_ | W068 with ABD1_29160 deleted, using suicide plasmid pGEM-sacB-△tonB_2_; Kan^R^ | This study |
| △xcpS | W068 with ABD1_03410 deleted, using suicide plasmid pGEM-sacB-△xcpS ; Kan^R^ | This study |
| △xcpU | W068 with ABD1_15710 deleted, using suicide plasmid pGEM-sacB-△xcpU ; Kan^R^ | This study |
| △xcpV | W068 with ABD1_15720 deleted, using suicide plasmid pGEM-sacB-△xcpV ; Kan^R^ | This study |
| △xcpW | W068 with ABD1_15730 deleted, using suicide plasmid pGEM-sacB-△xcpW ; Kan^R^ | This study |
| pilF^C^ | △pilF obtain recombination by suicide plasmid pGEM- pilF; kan^R^, Tet^R^ | This study |
| pilQ^C^ | △pilQ obtain recombination by suicide plasmid pGEM- pilQ; kan^R^, Tet^R^ | This study |
| tsaP^C^ | △ tsaP obtain recombination by suicide plasmid pGEM- tsaP ; kan^R^, Tet^R^ | This study |
| pilM^C^ | △pilM obtain recombination by suicide plasmid pGEM- pilM; kan^R^, Tet^R^ | This study |
| pilN^C^ | △pilNobtain recombination by suicide plasmid pGEM- pilN; kan^R^, Tet^R^ | This study |
| pilO^C^ | △pilO obtain recombination by suicide plasmid pGEM- pilO; kan^R^, Tet^R^ | This study |
| pilP^C^ | △pilP obtain recombination by suicide plasmid pGEM- pilP; kan^R^, Tet^R^ | This study |
| pilB^C^ | △pilB obtain recombination by suicide plasmid pGEM- pilB; kan^R^, Tet^R^ | This study |
| pilC^C^ | △pilC obtain recombination by suicide plasmid pGEM- pilC; kan^R^, Tet^R^ | This study |
| pilT^C^ | △pilT obtain recombination by suicide plasmid pGEM- pilT; kan^R^, Tet^R^ | This study |
| pilD^C^ | △pilD obtain recombination by suicide plasmid pGEM- pilD; kan^R^, Tet^R^ | This study |
| pilE^C^ | △pilE obtain recombination by suicide plasmid pGEM- pilE; kan^R^, Tet^R^ | This study |
| pilY2^C^ | △pilY2 obtain recombination by suicide plasmid pGEM- pilY2; kan^R^, Tet^R^ | This study |
| pilY1^C^ | △pilY1 obtain recombination by suicide plasmid pGEM- pilY1; kan^R^, Tet^R^ | This study |
| pilX^C^ | △pilX obtain recombination by suicide plasmid pGEM- pilX; kan^R^, Tet^R^ | This study |
| pilW^C^ | △pilW obtain recombination by suicide plasmid pGEM- pilW; kan^R^, Tet^R^ | This study |
| pilV^C^ | △pilV obtain recombination by suicide plasmid pGEM- pilV; kan^R^, Tet^R^ | This study |
| fimU^C^ | △ fimU obtain recombination by suicide plasmid pGEM- fimU ; kan^R^, Tet^R^ | This study |
| tonB_2_^C^ | △tonB_2_ obtain recombination by suicide plasmid pGEM- tonB_2_; kan^R^, Tet^R^ | This study |
| crp^C^ | △ crp obtain recombination by suicide plasmid pGEM- crp ; kan^R^, Tet^R^ | This study |
| comEA^C^ | △ comEA obtain recombination by suicide plasmid pGEM- comEA ; kan^R^, Tet^R^ | This study |
| comA^C^ | △ comA obtain recombination by suicide plasmid pGEM- comA ; kan^R^, Tet^R^ | This study |
| comF^C^ | △ comF obtain recombination by suicide plasmid pGEM- comF ; kan^R^, Tet^R^ | This study |
| priA^C^ | △ priA obtain recombination by suicide plasmid pGEM- priA ; kan^R^, Tet^R^ | This study |
| recA^C^ | △ recA obtain recombination by suicide plasmid pGEM- recA ; kan^R^, Tet^R^ | This study |
| xcpW^C^ | △ xcpW obtain recombination by suicide plasmid pGEM- xcpW ; kan^R^, Tet^R^ | This study |

1. Hu, Y., He, L., Tao, X., Meng, F., and Zhang, J. (2019). High DNA Uptake Capacity of International Clone II Acinetobacter baumannii Detected by a Novel Planktonic Natural Transformation Assay. *Front Microbiol* 10**,** 2165. doi: 10.3389/fmicb.2019.02165.
