# Supplementary material for: Natural Transformation in Acinetobacter baumannii W068: A Genetic Analysis Reveals the Involvements of the CRP, XcpV, XcpW, TsaP, and TonB2
Source: Front Microbiol. 2022 Jan 20;12:738034. doi: 10.3389/fmicb.2021.738034 (PMC8811193; doi:10.3389/fmicb.2021.738034)
Supplement: Supplementary file 2 [file Table_2.DOCX]

Table 2S Primers used in this study

| **Primers*** | **Sequence** |
| --- | --- |
| **For construction of knockout suicide plasmids** | |
| sacB-F | TGCGTAACTAACTTGCCATCT |
| sacB-R | GGCATTTTCTTTTGCGTTTT |
| kan-F | ATTCCGGGGATCCGTCGACC |
| kan-R | GTGTAGGCTGGAGCTGCTTCG |
| pGM-sacB-F | TGGATCCGAGCTCGGTAGCCCAATTCGCCCTATAGTG |
| pGM-sacB-R | TGCGTAACTAACTTGCCATCT |
| pilF-UF | TACCGAGCTCGGATCCACGTAGGTGAAGGTTCTGGCT |
| pilF-UR | GGTCGACGGATCCCCGGAATAGCAACCCCCATACATAACA |
| pilF-DF | CGAAGCAGCTCCAGCCTACACATCCTAATTCACAGCAGCCA |
| pilF-DR | GGCAAGTTAGTTACGCAGCCAACATCGCAAGTTTCGGTA |
| pilQ-UF | TACCGAGCTCGGATCCACAACCAATTGCCATAGAAGC |
| pilQ-UR | GGTCGACGGATCCCCGGAATCCCATAGAAAACTGACGGAA |
| pilQ-DF | CGAAGCAGCTCCAGCCTACACTGCTGATTTTCGTTACACCAC |
| pilQ-DR | AGTTAGTTACGCAGCTCGCAACCGTTTTACCTAGA |
| tsaP-UF | TACCGAGCTCGGATCCAGATGGTCAGGTACTTGCGC |
| tsaP-UR | GGTCGACGGATCCCCGGAATAATTATGTGTGCAGTTGCGTT |
| tsaP-DF | CGAAGCAGCTCCAGCCTACACGGTTGGTTCAAGCATTCAGC |
| tsaP-DR | AGTTAGTTACGCAGCTGTTGCTGACTTTGCCATTG |
| pilM-UF | TACCGAGCTCGGATCCAAAGTTGAAGCATTCGGCCTA |
| pilM-UR | GGTCGACGGATCCCCGGAATTCGACACCCATTAACCCCT |
| pilM-DF | CGAAGCAGCTCCAGCCTACACGGCTTGGCTTTAAGGAGTTT |
| pilM-DR | GGCAAGTTAGTTACGCAGCAACAATGAAATACCCCAAAGC |
| pilN-UF | TACCGAGCTCGGATCCAATGCTTTGATGCCTTTACCC |
| pilN-UR | GGTCGACGGATCCCCGGAATTCCTTAAAGCCAAGCCACAT |
| pilN-DF | CGAAGCAGCTCCAGCCTACACGTGACCACAACGGATGATTC |
| pilN-DR | GGCAAGTTAGTTACGCAGCAATCGGTGCTGGTTCAATAG |
| pilO-UF | TACCGAGCTCGGATCCAGCTGGTGGAAATGCGAATA |
| pilO-UR | GGTCGACGGATCCCCGGAATGCCCCCACACTTTCACCT |
| pilO-DF | CGAAGCAGCTCCAGCCTACACTGTTCAGCCGAAATAGGAGAG |
| pilO-DR | GGCAAGTTAGTTACGCAGCCGTGAACGCTGGTCATCTG |
| pilP-UF | TACCGAGCTCGGATCCAGTTGTGACTGTCGATTTGGG |
| pilP-UR | GGTCGACGGATCCCCGGAATATAACTCTCCTATTTCGGCTGA |
| pilP-DF | CGAAGCAGCTCCAGCCTACACTTGGTCTTGATTGGGCCT |
| pilP-DR | GGCAAGTTAGTTACGCAGCAACATTTTTATCTTGGGGACG |
| fimV-UF | TACCGAGCTCGGATCCATCATCGTTCTTGATCCGGTT |
| fimV-UR | GGTCGACGGATCCCCGGAATAATCCATGACCTGATCAGTCG |
| fimV-DF | CGAAGCAGCTCCAGCCTACACGGCACAGCAACAAACTCACA |
| fimV-DR | GGCAAGTTAGTTACGCAGCACTCTAAATCGGCCAGCTCA |
| pilB-UF | TACCGAGCTCGGATCCAGAGCCTGCTGGTGTAAGTTG |
| pilB-UR | GGTCGACGGATCCCCGGAATCTGCCTGCCTATGTTAATGTG |
| pilB-DF | CGAAGCAGCTCCAGCCTACACCAAGGGGTGACCTCTTTACAA |
| pilB-DR | GGCAAGTTAGTTACGCAGCCAATAAGTGGAACACCTGCG |
| pilC-UF | TACCGAGCTCGGATCCAGAACTCCCTCCCGACTTTG |
| pilC-UR | GGTCGACGGATCCCCGGAATTTGACAGCCATTAGATTATCCTT |
| pilC-DF | CGAAGCAGCTCCAGCCTACACATTTTAGGGGTGCTCGTAGG |
| pilC-DR | GGCAAGTTAGTTACGCAGCAAGCCATCTTAACCTCCCAA |
| pilT-UF | atcaaGCTTGGTACCGAGCTCGGATCCAGTCGTACCTGTCTGCATCCA |
| pilT-UR | GGTCGACGGATCCCCGGAATGCTTTCACCGAGGCTTACC |
| pilT-DF | CGAAGCAGCTCCAGCCTACACAAAGGCTTAGTCGCTCGC |
| pilT-DR | tttgaAGATGGCAAGTTAGTTACGCAGCTCTGGTGTTGGAATGAGCTG |
| pilU-UF | atcaaGCTTGGTACCGAGCTCGGATCCAGCGCCGTGACTATGAAGAA |
| pilU-UR | GGTCGACGGATCCCCGGAATTTGAGCAAGTCATTAAAATCCA |
| pilU-DF | CGAAGCAGCTCCAGCCTACACCCCGATCAATTATCAGACGG |
| pilU-DR | tttgaAGATGGCAAGTTAGTTACGCAGCAATGCTGACCATGTGCTGC |
| pilD-UF | TACCGAGCTCGGATCCAAAAGGTGAAGTCGAAGGTGG |
| pilD-UR | GGTCGACGGATCCCCGGAATTTGCATTATACAACTGAGCCC |
| pilD-DF | CGAAGCAGCTCCAGCCTACACTTTGGGAGGTTAAGATGGCT |
| pilD-DR | AGTTAGTTACGCAGCATGGCGTGTTTGTCGTTATT |
| pilE-UF | TACCGAGCTCGGATCCATTTATGACCCTCAAGGAACG |
| pilE-UR | GGTCGACGGATCCCCGGAATAAACCATTCTTCACTGCCAA |
| pilE-DF | CGAAGCAGCTCCAGCCTACACATTCAAAACTGCGGATAACTCA |
| pilE-DR | GGCAAGTTAGTTACGCAGCGTAAATGCGACCAGCTTCAA |
| pilY2-UF | TACCGAGCTCGGATCCATCAGAGTTGTGCGTTTAGCG |
| pilY2-UR | GGTCGACGGATCCCCGGAATCATACCAGCGCACAGGAAG |
| pilY2-DF | CGAAGCAGCTCCAGCCTACACTTGCGTTGTCAAAGTTTAACAG |
| pilY2-DR | GGCAAGTTAGTTACGCAGCATACACCCAGAGCCACCCA |
| pilY1-UF | TACCGAGCTCGGATCCATATTTCTGTCGGGGTTTTGG |
| pilY1-UR | GGTCGACGGATCCCCGGAATGCCATGTGAATATCATTGAGC |
| pilY1-DF | CGAAGCAGCTCCAGCCTACACAGAACGGAACAGGTGAGTGG |
| pilY1-DR | GGCAAGTTAGTTACGCAGCTGAGTTATCCGCAGTTTTGAA |
| pilX-UF | TACCGAGCTCGGATCCAATTGGAGGGCTTTTATCGAG |
| pilX-UR | GGTCGACGGATCCCCGGAATTGTTTCCCCCATTGCATT |
| pilX-DF | CGAAGCAGCTCCAGCCTACACTGACTGAAATCACGGCACC |
| pilX-DR | GGCAAGTTAGTTACGCAGCTCGCCATCCGATAAGAAGT |
| pilW-UF | TACCGAGCTCGGATCCAAGAAGCGAAAAGTCAGGCAA |
| pilW-UR | GGTCGACGGATCCCCGGAATAGCCATTTAATATGCCTCCA |
| pilW-DF | CGAAGCAGCTCCAGCCTACACCTTTACGCAATGCAATGGG |
| pilW-DR | GGCAAGTTAGTTACGCAGCCATTATGAACCTGGTGCCG |
| pilV-UF | TACCGAGCTCGGATCCAGTGAGCTTGCTGAGCGTATG |
| pilV-UR | GGTCGACGGATCCCCGGAATTAACAATTCTCCTCGGCCTT |
| pilV-DF | CGAAGCAGCTCCAGCCTACACAACTTGCTTAATGATGGAGGC |
| pilV-DR | GGCAAGTTAGTTACGCAGCGCATTATTTGTAGAACGGGC |
| fimU-UF | TACCGAGCTCGGATCCAATGACTTACGCCAAAGAGGG |
| fimU-UR | GGTCGACGGATCCCCGGAATTCCCCAGAACCCCAAATAA |
| fimU-DF | CGAAGCAGCTCCAGCCTACACGGTTTTTAAGGCCGAGGAGA |
| fimU-DR | GGCAAGTTAGTTACGCAGCTGGCATTTGTTGCTGTTGA |
| comEA-UF | TACCGAGCTCGGATCCAAACCCCGCTGATAAAATACC |
| comEA-UR | GGTCGACGGATCCCCGGAATTCATGCTGTATTGCATGTGG |
| comEA-DF | CGAAGCAGCTCCAGCCTACACTCAATTATCATTTCCTGCGC |
| comEA-DR | GGCAAGTTAGTTACGCAGCCAACGCTCTAAAAATGGCTG |
| comA-UF | TACCGAGCTCGGATCCACTTGGCAACTATTTATCCGG |
| comA-UR | GGTCGACGGATCCCCGGAATGGCAAAATTAAAGCCCAACT |
| comA-DF | CGAAGCAGCTCCAGCCTACACTTTACATGACCGACGTTTGG |
| comA-DR | GGCAAGTTAGTTACGCAGCTTTACATGACCGACGTTTGG |
| comF-UF | TACCGAGCTCGGATCCAATTATTGATGAGCAGCACCG |
| comF-UR | GGTCGACGGATCCCCGGAATTATGCGTATCGAGGAGCTTC |
| comF-DF | CGAAGCAGCTCCAGCCTACACGCCTAGCTGCTGCTTCATCT |
| comF-DR | GGCAAGTTAGTTACGCAGCGTCATTGGGGTAATTCCTCG |
| priA-UF | TACCGAGCTCGGATCCAAAAGCATAATGTCCCGCAGT |
| priA-UR | GGTCGACGGATCCCCGGAATTGGTCATAGAGGCTCATTTCG |
| priA-DF | CGAAGCAGCTCCAGCCTACACGATGTTGACCCGCAAGAATT |
| priA-DR | GGCAAGTTAGTTACGCAGCGCTTCAAGCTCATGACGGA |
| dprA-UF | TACCGAGCTCGGATCCAATAGCGTTCCGGTTATTCCA |
| dprA-UR | GGTCGACGGATCCCCGGAATAAAGGTACATCCAGCATTAATCAT |
| dprA-DF | CGAAGCAGCTCCAGCCTACACTGTATGCAACAATCAGGGCT |
| dprA-DR | GGCAAGTTAGTTACGCAGCTGCCACATAATAGCGACCAC |
| recA-UF | TACCGAGCTCGGATCCAGCTGACTGTGCAAGCCTAAA |
| recA-UR | GGTCGACGGATCCCCGGAATCATCCATCTCAAAAACCTCAA |
| recA-DF | CGAAGCAGCTCCAGCCTACACGTCATAAAACGCCCTTCGG |
| recA-DR | GGCAAGTTAGTTACGCAGCAGCCACCTTGACACAGGAATA |
| comM-UF | TACCGAGCTCGGATCCATCATTCGTGTCCCCCAAC |
| comM-UR | GGTCGACGGATCCCCGGAATTTGTTCCATTATGGTGCTTCA |
| comM-DF | CGAAGCAGCTCCAGCCTACACGGTCATTCAAAGTCCTCATCTT |
| comM-DR | GGCAAGTTAGTTACGCAGCGGCTTTCAAGTGGTTTCTGTC |
| xcpS-UF | TACCGAGCTCGGATCCATTTGGTCTTCATTGAGCGTG |
| xcpS-UR | GGTCGACGGATCCCCGGAATTGCAGGCATGGTAATGTTCT |
| xcpS-DF | CGAAGCAGCTCCAGCCTACACGCGGTTATGCTGCCAATC |
| xcpS-DR | GGCAAGTTAGTTACGCAGCCATCGCCCAATGAAACTGA |
| xcpU-UF | TACCGAGCTCGGATCCAGGACTGGGAAACAGCAAAAG |
| xcpU-UR | GGTCGACGGATCCCCGGAATTGCTTGTTTGGGTAAGGCTA |
| xcpU-DF | CGAAGCAGCTCCAGCCTACACGAAATCTAAAGGCTTTACCCTCA |
| xcpU-DR | GGCAAGTTAGTTACGCAGCCCATTTTGTGACTTCTTGCG |
| xcpV-UF | TACCGAGCTCGGATCCACCAAGTGAGAAAGCAACGC |
| xcpV-UR | GGTCGACGGATCCCCGGAATTGATCTATTTGTATTTCCGAACC |
| xcpV-DF | CGAAGCAGCTCCAGCCTACACTGATCCGGATAAGGGAAAAG |
| xcpV-DR | GGCAAGTTAGTTACGCAGCTGTAGCCAAGGCAACCATAA |
| xcpW-UF | TACCGAGCTCGGATCCAGGCATTAGCCTTACCCAAAC |
| xcpW-UR | GGTCGACGGATCCCCGGAATTCATTATTTTGCTTTCACTGGAT |
| xcpW-DF | CGAAGCAGCTCCAGCCTACACTCAGGGTGACTTATCGCTTTC |
| xcpW-DR | GGCAAGTTAGTTACGCAGCGCACTAGTAAACTGTCGCTTCC |
| crp-UF | TACCGAGCTCGGATCCACTACGCCACCATCTGAAAGC |
| crp-UR | GGTCGACGGATCCCCGGAATAGTCATGCTTGATATGTTCCGA |
| crp-DF | CGAAGCAGCTCCAGCCTACACCACTGACGAAGACTATGATGACG |
| crp-DR | GGCAAGTTAGTTACGCAGCGACTATACGGATTTTGCACACTC |
| tonB2-UF | TACCGAGCTCGGATCCAGGCCTTTTTAGCAAACCAAC |
| tonB2-UR | GGTCGACGGATCCCCGGAATCGCAAAATGGATGAGCAAC |
| tonB2-DF | CGAAGCAGCTCCAGCCTACACGGCGTTTTGATCCAGCTG |
| tonB2-DR | GGCAAGTTAGTTACGCAGCTCTTGACCCGTTTAGGTTTTG |
| **For detection of knockout mutants and the single-crossover event** | |
| pilF-F | TTTGGCAAGTGGTTGTCAGA |
| pilF-R | TTGAAATGTAAGAGTCGCGG |
| pilQ-F | GCAGGTCAGGGCACAGAAA |
| pilQ-R | GGTCCCTCCACCAGAAGTATG |
| tsaP-F | TACAACCACAAGTTCGCGTC |
| tsaP-R | CGAATAATCTTGCCACCATC |
| pilM-F | GAGCTCTCTGTCAAGAACGGT |
| pilM-R | TGCCATTTTGCATGACAGAT |
| pilN-F | TAATTTACTGCCTTGGCGC |
| pilN-R | CTTCAATCGTAAACTTATCTCCG |
| pilO-F | TCTCGATAAATTCCTTCAGCAA |
| pilO-R | CGCTTCTATGGCAATTGGT |
| pilP-F | CTCTTTATGTTTTGGCGTGTACT |
| pilP-R | AATCTGCTGATCCGGTGTCT |
| fimV-F | CGCAACCCGTCTTCAACA |
| fimV-R | CGGGCCTTTATAAGCGCT |
| pilB-F | CATCTCCAAAATTTACGGGG |
| pilB-R | AACCTGTTGGTCCGGTAATT |
| pilC-F | GGCGTAAAAATCAAGGGAGA |
| pilC-R | CTCGCATAGCAAACTGAAGC |
| pilT-F | GAGCTACTCGCCTTCTCTGTG |
| pilT-R | GCCATTCTTTTTAAGCAGGG |
| pilU-F | GGTTGAAAAAAAATCATCGGA |
| pilU-R | GAATAATACGGTCGAGCGCT |
| pilD-F | CAAGACATCATTGCGTATTTTATT |
| pilD-R | GCTGAGTTGGGTGAGGTATAAA |
| pilE-F | TTGGCAGTGAAGAATGGTTT |
| pilE-R | GCACCTTTTGCCCAAAATT |
| pilY2-F | GGTCGTGATTGTTATCGTTGC |
| pilY2-R | TCCTGTCGTCGTATCATCAGTAAT |
| pilY1-F | CCTACAGCAGGTAAAAAAACAAT |
| pilY1-R | TACCAAAACCGACAAATGCT |
| pilX-F | TATTTCTGTCGGGGTTTTGG |
| pilX-R | GAGGTAGATTTGAACCCGGA |
| pilW-F | GAACTTATAGTCGCCTTAGCTTTAG |
| pilW-R | AGACCTTTCCAGTGGTTATTCA |
| pilV-F | ACTCGAAGTTTTGGTTGCCT |
| pilV-R | AAGACAAATAGGCACTGGCG |
| fimU-F | CACCTTGGTCGAGCTAATGG |
| fimU-R | ATTTGCCACTACTCCATTTGC |
| comEA-F | GGGAGTAATTTCACCTTCGG |
| comEA-R | GTATTCTACAATCGCTTGAGCC |
| comA-F | TCTATTGGGGTGGATTGGC |
| comA-R | GGTTGATAATAATGTTCGTAGTGC |
| comF-F | TTGCTGTCCCCTTGCCTAC |
| comF-R | GCTAGGCAACTGGCATGAAT |
| priA-F | CAGGCCATGCGCTTTTAC |
| priA-R | ATGCTCTTCAACCTTGGCTG |
| dprA-F | TGAAAAAGCCACTCAACCTG |
| dprA-R | ATCGCTATTGTGCGCTCTAA |
| recA-F | GCACGCAAACTTGGTGTAGA |
| recA-R | GAACCAACAATTTCATCGCC |
| comM-F | GTAAGACCCGTATCAGGCG |
| comM-R | GGAGAACACTGACAACGGCT |
| xcpS-F | TTCAGGGAAGCAGCAAAAG |
| xcpS-R | CCAGCATAAGCGGTTCAAG |
| xcpU-F | CGAATCTACCATTTCAATCACA |
| xcpU-R | TAGCTTCTCCATTGCCAAAC |
| xcpV-F | GAAGTTATGGTTGCTTTGGC |
| xcpV-R | ATCCGGATCATACAAACTCACT |
| xcpW-F | CTCGATTAACTCGCGCCTC |
| xcpW-R | ATTTTGTGACTTCTTGCGGAG |
| crp-F | TCCTGGTGACTTCTTTGGGG |
| crp-R | CCGTCAGTTTGAATCATACCTTG |
| tonB-F | GTCGAGTAACTCTCCAGCGC |
| tonB-R | TGCCTTTGCTGCTTCATCTA |
| plasmid-F | GGTACCGAGCTCGGATCCA |
| Plasmid-R | AGATGGCAAGTTAGTTACGCAGC |
| **For construction of complementing plasmids** | |
| TET-F | TTCTCATGTTTGACAGCTTATCAT |
| TET-R(C) | GGCAAGTTAGTTACGCAGCCATTCAGGTCGAGGTGGC |
| pGM-F | TGGATCCGAGCTCGGTAGCCCAATTCGCCCTATAGTG |
| pGM-R | GCTGCGTAACTAACTTGCCGTCACCTAAATAGCTTGGCGTA |
| pilF-CR | TAAGCTGTCAAACATGAGAATTTATTTCCATACGGCCTCA |
| pilQ-CR | TAAGCTGTCAAACATGAGAACGCCACCTATTTCAATTGC |
| tsaP-CR | TAAGCTGTCAAACATGAGAAAAAGGTACATCCAGCATTAATCA |
| pilM-CR | TAAGCTGTCAAACATGAGAAGTTGCCATTAATCAAAACTCC |
| pilN-CR | TAAGCTGTCAAACATGAGAATTGACTCATTTAGCAGCCCC |
| pilO-CR | TAAGCTGTCAAACATGAGAATCCTATTTCGGCTGAACAGT |
| pilP-CR | TAAGCTGTCAAACATGAGAATAAATTTTAGGGTGCAGGCC |
| pilB-CR | TAAGCTGTCAAACATGAGAATCTTATTCACTGGTGACACGG |
| pilC-CR | TAAGCTGTCAAACATGAGAATAGGCTCACGAGTGCAACAG |
| pilT-CR | TAAGCTGTCAAACATGAGAATGTATTCTCGCTATTTTGTTGTTT |
| pilD-CR | TAAGCTGTCAAACATGAGAACTTGGATTCCTTGGGATTCA |
| pilE-CR | TAAGCTGTCAAACATGAGAATTGAGTTATCCGCAGTTTTGA |
| pilY2-CR | TAAGCTGTCAAACATGAGAATCACTGCCAAACCTCACTGT |
| pilY1-CR | TAAGCTGTCAAACATGAGAACTAGCGATAGCGCTCATACC |
| pilX-CR | TAAGCTGTCAAACATGAGAATTATGAACCTGGTGCCGTG |
| pilW-CR | TAAGCTGTCAAACATGAGAAGGTCATAGTGTTTCCCCCAT |
| pilV-CR | TAAGCTGTCAAACATGAGAAAGCCATTTAATATGCCTCCA |
| fimU-CR | TAAGCTGTCAAACATGAGAAGCATTAACAATTCTCCTCGG |
| crp-CR | TAAGCTGTCAAACATGAGAACGAGTAATTATTCTTCGTCATCAT |
| tonB-CR | TAAGCTGTCAAACATGAGAACGATTAAGAACGCAGTCAGC |
| comEA-CR | TAAGCTGTCAAACATGAGAATTTATAAGGCTAAACGCGATT |
| comA-CR | TAAGCTGTCAAACATGAGAAAAGCTTTAAAAACCTCTACTCAAC |
| comF-CR | TAAGCTGTCAAACATGAGAACCGTGGTGTGAGCAGAACTT |
| priA-CR | TAAGCTGTCAAACATGAGAATGCCCCGAATGTATTGATCT |
| recA-CR | TAAGCTGTCAAACATGAGAACCGAAGGGCGTTTTATGA |
| xcpW-CR | TAAGCTGTCAAACATGAGAAAACGGCCATTATGAATTACCT |

* For each gene, primer labeled with “-UF” were paired with the primer labeled with “-UR” to amplify the upstream region; Primer marked by “-DF” were paired with the one marked by “-DR” to amplify the downstream region; Primers labeled with “-CR” were paired with primers labeled with “-UF” of each gene to construct the complement plasmid.
